# The Impact of Chatbot Type and Normative Messaging on Chatbot Usage Intention Based on the Health Technology Acceptance Model: Randomized Controlled Trial

**DOI:** 10.2196/97773

**Published:** 2026-09-15

**Authors:** Yi Liao, Anne Madeo, Caitlin G Allen, Melissa K Frey, Whitney Maxwell, Chelsey Schlechter, Ravi N Sharaf, Kensaku Kawamoto, Guilherme Del Fiol, Kimberly A Kaphingst

**Affiliations:** 1 Department of Communication, University of Utah, 255 S Central Campus Dr, Salt Lake City, UT, 84112, United States, 1 801-581-6889; 2 Huntsman Cancer Institute, Salt Lake City, UT, United States; 3 Center for Health Optimization and Implementation Research (CHOIR), VA Bedford and VA Boston Healthcare Systems, Boston, MA, United States; 4 School of Medicine, Wake Forest University, Winston-Salem, NC, United States; 5 Weill Cornell Medicine, New York City, NY, United States; 6 Department of Population Health Sciences, University of Utah, Salt Lake City, UT, United States; 7 Department of Biomedical Informatics, University of Utah, Salt Lake City, UT, United States

**Keywords:** digital health, technology acceptance, social norm, chatbot, AI

## Abstract

**Background:**

Digital health tools, such as health chatbots, may improve access to scalable health support, but adoption remains inconsistent. Existing models do not fully integrate technology acceptance factors with health motivation factors relevant to digital health use.

**Objective:**

This study proposed and tested the health technology acceptance model and examined whether normative message framing and chatbot type were associated with health motivation, technology acceptance, and intention to use a health chatbot.

**Methods:**

In October 2025, we conducted a 4 × 2 between-participants online experiment with 1000 US adults recruited from a nationally representative YouGov panel. Participants were randomized to 1 of 8 conditions varying norm message type (self-oriented, peer-oriented, expert-oriented, or family-oriented) and chatbot type (AI-powered or rule-based) in a cancer prevention and genetic risk information scenario. Outcomes included descriptive norms, injunctive norms, perceived susceptibility, perceived severity, perceived benefits, self-efficacy, perceived ease of use, trust, privacy concerns, and usage intention. Data were analyzed using a multivariate ANOVA with Bonferroni-adjusted post hoc tests and multiple linear regression.

**Results:**

Peer-oriented and family-oriented messages produced higher usage intention than expert-oriented messages, and peer-oriented messages also increased descriptive norms, injunctive norms, self-efficacy, and trust. AI-powered chatbots were associated with higher usage intention (*P*=.02) and greater trust (*P*=.008) than rule-based chatbots. In regression analyses, the model explained 50.8% of the variance in usage intention. Usage intention was positively associated with descriptive norms (β=0.087; *P*=.003), injunctive norms (β=0.078; *P*=.009), perceived susceptibility (β=0.051; *P*=.03), perceived benefits (β=0.253; *P*<.001), and trust (β=0.33; *P*<.001), and negatively associated with perceived severity (β=–0.047; *P*=.049) and privacy concerns (β=−0.11; *P*<.001). Perceived ease of use and self-efficacy were not significant predictors.

**Conclusions:**

The health technology acceptance model was a useful framework for explaining the intention to use a health chatbot by combining technology acceptance and health motivation constructs. Both social design features and chatbot design features shaped adoption-related beliefs, with peer-oriented and family-oriented framing and AI-powered chatbots showing particular promise. Trust and privacy concerns remained central determinants of intended use.

## Introduction

### Background

As health care systems globally face increasing burdens, including provider shortages and rising chronic disease prevalence, and as technology advances such as in AI, digital health technologies have emerged as a critical solution for scalable, accessible health management [1-5]. Among these technologies, health chatbots, also known as health conversational agents, have gained significant traction, evolving from simple administrative tools to sophisticated agents capable of delivering personalized medical information, symptom assessment, and behavioral support [6-9]. Although health chatbots are widely deployed, users’ willingness to adopt and engage with these systems varies substantially [10-13]. Usage intention remains a critical outcome because it represents the immediate psychological precursor to actual adoption and sustained use of health technologies [7,14,15]. Understanding what drives usage intention, therefore, has both theoretical and practical significance for the design of effective digital health interventions.

To date, theoretical frameworks explaining health technology adoption have remained fragmented. The technology acceptance model (TAM) has long served as the dominant paradigm; indeed, prior reviews indicate that between 1999 and 2017, telemedicine applications that constituted the information and communication technology domain were most frequently examined using TAM [16]. TAM, originating in information systems research, focuses primarily on usability-related perceptions such as ease of use and usefulness [17]. However, because it was not developed specifically for health contexts, TAM often gives limited attention to the cognitive, motivational, and risk-related factors that shape health-related use intention and behavior. Consistent with this limitation, most telemedicine studies have called for substantial extensions to the original model, suggesting that no single optimal version of TAM has been established for health service contexts [16,18].

Subsequent extensions have broadened TAM’s scope. TAM3 added determinants such as computer self-efficacy and computer anxiety [19], while unified theory of acceptance and use of technology 2 introduced hedonic motivation, price value, and habit [20]. Nevertheless, these models remain primarily centered on system usability and technology-related perceptions rather than on users’ perceived disease risk, disease severity, or expected health benefits. Within health informatics, researchers have further extended TAM by incorporating constructs from the health belief model, the theory of planned behavior, and related concepts such as trust and privacy [21-23]. These studies demonstrate the value of combining usability beliefs with health-relevant and contextual factors, but they often do so by appending additional constructs to a usability-centered framework. In contrast, some studies have used health behavior theories, such as the health belief model and the theory of planned behavior, to explore usage; these theories emphasize perceptions of disease threat and health motivation [14,21,22,24], but they overlook the technological considerations and usability barriers inherent in digital health tools.

To bridge this theoretical divide, this study proposes the health technology acceptance model (HTAM). This framework integrates the cognitive evaluations of system characteristics found in TAM with the motivational drivers from health behavior theories. HTAM conceptualizes usage intention as the outcome of technology acceptance and health motivation, both of which are shaped by external variables. HTAM positions external variables such as the characteristics of the health technology, message features, and demographic characteristics of users as factors that can be associated with different technology acceptance and health motivation variables. To empirically test HTAM, this study used chatbot technology as a timely case study, operationalizing the external variables through 2 independent variables: norm message type (self-oriented, peer-oriented, expert-oriented, and family-oriented) and chatbot type (AI-powered vs rule-based). Instead of treating these features as the primary phenomena under investigation, they serve as experimental inputs designed to trigger the internal acceptance and motivation mechanisms defined by HTAM. This approach allows the study to demonstrate how varied external factors jointly activate the cognitive and behavioral paths that ultimately shape usage intention.

### HTAM

#### Proposed HTAM

**Figure 1.**
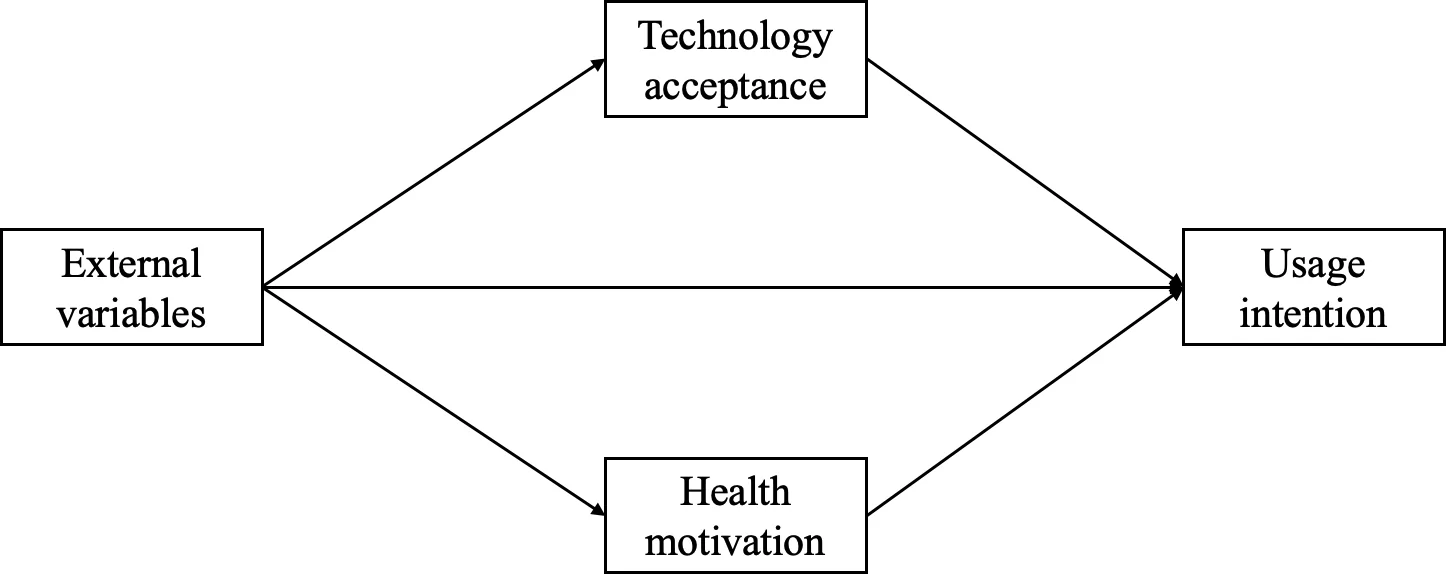
Conceptual model.

HTAM (Figure 1) is proposed as an integrative framework that bridges technology acceptance research and health behavior theories. Traditional models such as TAM focus primarily on cognitive evaluations of system characteristics [17], while health behavior theories emphasize perceptions of risk, benefits, and social influence [24,25]. Although some theories, like the unified theory of acceptance and use of technology, already consider the role that social influence plays in technology acceptance, HTAM argues that neither perspective alone is sufficient for explaining engagement with health technologies, which are inherently both technological systems and tools that support personal health.

#### Technology Acceptance

In health contexts, however, perceived usefulness and perceived health benefits can be difficult to separate. In TAM’s original settings, a system’s usefulness is typically judged against an external task objective. For a preventive health chatbot, the task being supported is health management itself, such as understanding cancer risk or deciding whether to seek preventive care. As a result, judging the system useful and judging it beneficial for one’s health may reflect closely related evaluations. Conceptually, the 2 constructs remain distinguishable. Perceived usefulness concerns task-technology fit, or whether the system helps users accomplish a specific task, whereas perceived health benefits concern outcome efficacy, or whether using the information may help protect or improve one’s health. Yet these evaluations converge when the task itself is health management. Because the present study seeks to clearly distinguish technology acceptance from health motivation within HTAM, perceived usefulness is not treated as a separate acceptance variable. Instead, beliefs about the benefits of using a health technology are captured within the health motivation pathway as perceived health benefits.

Self-efficacy is incorporated into the technology acceptance pathway as a belief about one’s capability to successfully use a health technology. Although self-efficacy has been widely examined in health behavior research and was included in the TAM3 [19,22], within HTAM, it reflects confidence in navigating system functions, understanding outputs, and sustaining interaction with the technology. In digital health settings, where users must often articulate symptoms, interpret feedback, or manage ongoing interactions, self-efficacy plays a critical role in determining whether engagement feels feasible.

Trust and privacy concerns represent additional acceptance-related evaluations that extend beyond traditional TAM constructs [26-28]. Trust reflects beliefs about the accuracy, reliability, and integrity of the system, which is particularly important when technologies provide health-related information or recommendations [29,30]. Privacy concerns capture users’ apprehension regarding data collection, storage, and potential misuse of personal health information [29,31]. Prior research has treated trust and privacy concerns in different ways, including as antecedents to TAM variables, extensions of TAM, or direct predictors of acceptance and usage intentions [23,29,32,33].

#### Health Motivation

Health motivation variables commonly include perceptions of health risk, anticipated benefits of action, and social influence. Perceived susceptibility refers to beliefs about one’s likelihood of experiencing a health condition, while perceived severity reflects beliefs about how serious the condition itself is [25,34]. Together, these constructs capture perceived health threat, which has been shown to motivate information seeking and preventive behavior. Perceived benefits represent beliefs about the effectiveness of an action or tool in reducing health risks or improving health outcomes [35]. These benefits may include increased access to information, personalized guidance, or improved self-management. Social norms constitute an additional class of health motivation variables by signaling what behaviors are socially endorsed [36,37]. Descriptive norms convey perceptions of what most people do, whereas injunctive norms convey perceptions of what relevant others approve of or expect [38].

### Operationalizing External Variables: a Case Study

#### Selection of External Variables

The practical value of the HTAM rests on its ability to explain how specific external stimuli translate into internal beliefs. To empirically evaluate this framework, the current study selects 2 high-relevance external variables for manipulation: norm message type (a social communication variable) and chatbot type (a technological design variable).

#### Normative Messaging

Normative messages have been widely used in health communication to promote behavior change by highlighting social expectations and collective behavior [39-41]. Prior research demonstrates that individuals’ adoption of health behaviors is often guided by perceptions of what others commonly do (descriptive norms) and what important referent groups approve or expect (injunctive norms). Normative messaging has also been used in digital health interventions, including chatbot-based systems, to encourage engagement and sustained use [42,43].

In the present study, normative messaging is conceptualized as a social communication feature that shapes internal normative beliefs. Four norm orientations are examined: self-oriented (control condition), peer-oriented, expert-oriented, and family-oriented norms. These orientations reflect different social reference points that may vary in perceived relevance and persuasive strength. Prior research suggests that norms derived from proximal or identity-relevant groups may exert stronger motivational influence than abstract or distant sources [44-46]. By varying norm orientation, the current study examines how different social cues embedded in health chatbot messages shape descriptive and injunctive normative beliefs within HTAM.

#### Chatbot Type

The classification of chatbots into rule-based and AI-powered represents 2 different paradigms of human-computer interaction. Rule-based chatbots, also known as decision-tree or scripted chatbots, operate on predefined content and logic flows. They function as interactive flowcharts where every potential system response is hard-coded by developers or subject matter experts [47]. Prior research suggests that rule-based chatbots are often perceived as predictable, transparent, and controllable, which may reduce uncertainty during interaction and support user trust [48,49]. Their deterministic structure can also limit concerns related to unintended system behavior or opaque decision-making processes, potentially mitigating privacy and safety concerns [50]. However, because their responses are constrained by predefined rules and content, rule-based chatbots may offer limited personalization and adaptability, which can reduce perceived usefulness and engagement, particularly in complex or evolving health contexts [51-54]. Consequently, while users may trust the safety of a rule-based system, their intention to use it often wanes when tasks require high degrees of flexibility or complex problem-solving [55].

In contrast, AI-powered chatbots rely on AI approaches such as large language models (LLMs) to generate context-sensitive and adaptive responses based on probabilistic learning from large-scale data [56,57]. These systems are capable of processing natural language input more flexibly and providing personalized, conversational feedback that approximates human interaction [56,58]. Existing studies suggest that AI-powered chatbots may enhance perceived ease of use by supporting more natural dialogue and reducing the cognitive effort required to navigate system functions [7]. Their ability to tailor information and recommendations may also increase perceived benefits and strengthen users’ confidence in managing health-related tasks [59]. At the same time, the lack of transparency in LLM-based systems introduces unique challenges. Users may find it difficult to understand the mechanisms behind how the AI generates responses or how personal data are processed, which can complicate trust formation and heighten privacy concerns, particularly in health-related applications [58-60]. Ultimately, the decision to adopt AI-powered chatbots typically involves a strategic compromise: users accept reduced system interpretability in exchange for greater autonomy and adaptive capabilities [56].

### Research Questions and Hypotheses

**Figure 2.**
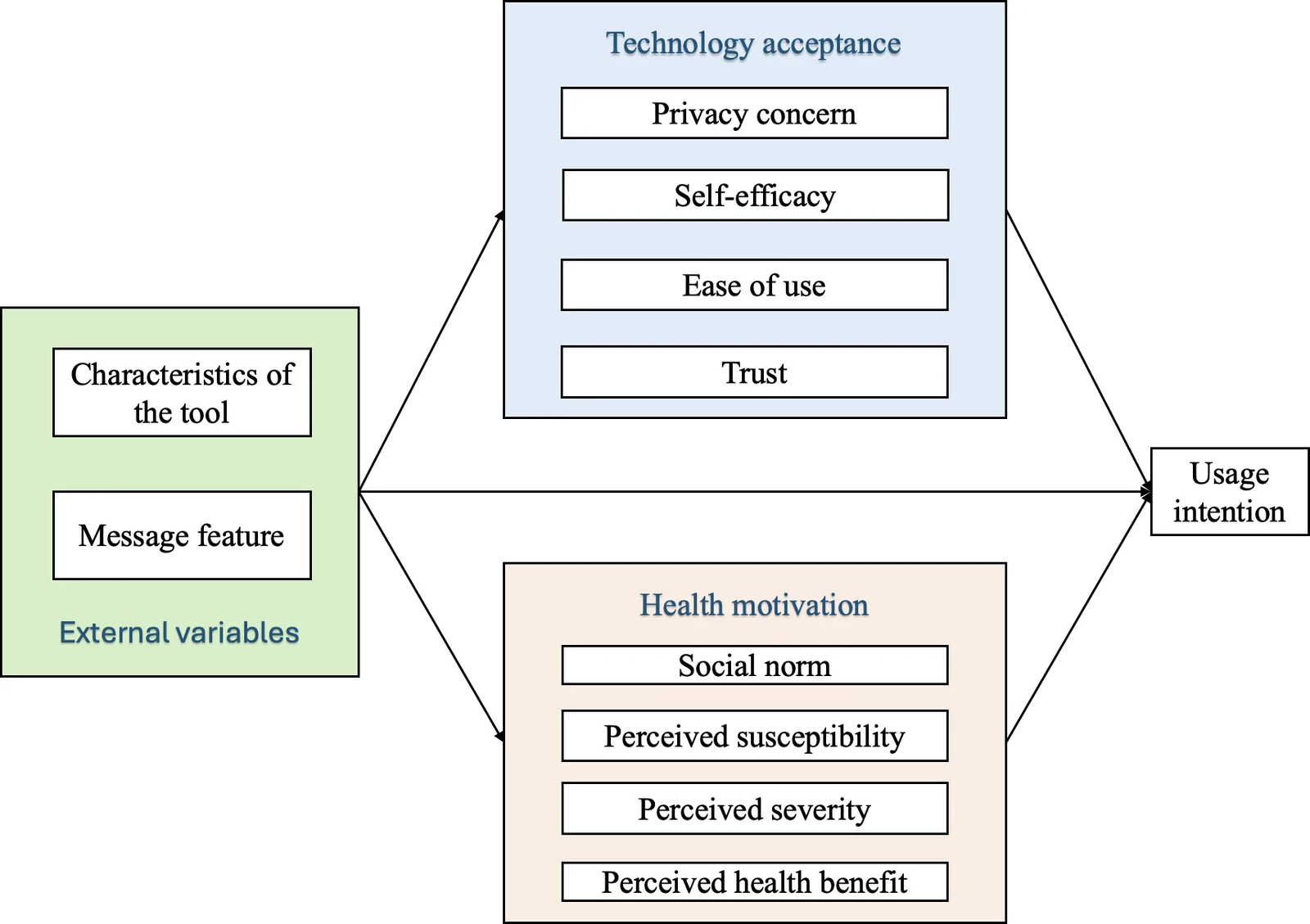
The current study.

Based on the proposed HTAM framework and the operationalization of external variables, the current study (Figure 2) proposes the following research question (RQ) and hypotheses:

RQ1: What is the relationship between design features (norm message type and chatbot type) and health motivation factors, technology acceptance factors, and usage intention?

H1: Health motivation factors (descriptive norm, injunctive norm, perceived susceptibility, perceived severity, and perceived benefits) will be positively associated with usage intention.

H2: Technology acceptance factors (ease of use, self-efficacy, and trust) will be positively associated with usage intention, whereas privacy concerns will be negatively associated with usage intention.

## Methods

### Study Design and Experimental Procedure

This study used a 4 × 2 between-participants factorial experimental design with 2 independent variables: norm message type (self-oriented, peer-oriented, expert-oriented, and family-oriented) and chatbot type (AI-powered vs rule-based). Participants were randomly assigned to one of 8 experimental conditions. Following exposure to the assigned stimulus, participants completed measures assessing health motivation factors, technology acceptance factors, and intention to use the chatbot in the provided scenarios.

The experimental scenario was situated within a cancer prevention and genetic risk information context. Cancer was selected as the focal health domain because it represents a high-salience condition with established theoretical relevance to perceived susceptibility, perceived severity, and perceived benefits. Grounding the chatbot in a specific health context enhances ecological validity and allows for a meaningful assessment of health motivation variables.

All stimuli and data are available on the Open Science Framework (OSF).

### Experimental Messages

To manipulate the normative appeal of the message, we compared a control condition with 3 treatment conditions featuring different social norms. The control condition presented a self-oriented message emphasizing personal health management and decision-making autonomy. The peer-oriented condition included information highlighting what similar others do. The expert-oriented condition included information emphasizing professional recommendations from the National Cancer Institute. The family-oriented condition emphasized family-related benefits and the protection of loved ones.

The chatbot type was also manipulated. The AI-powered condition described the chatbot as “powered by AI, which allows it to learn and provide personalized information.” The rule-based condition described it as “scripted by our health care team, which ensures consistency in the information provided.”

### Participants

The CONSORT-EHEALTH (Consolidated Standards of Reporting Trials of Electronic and Mobile Health Applications and Online Telehealth; Checklist 1) flow diagram in the Results section. Participants were recruited in October 2025 from a nationally representative sample using the YouGov panel [61]. Representativeness was achieved through YouGov’s standard sample-matching and weighting procedure: a target sample mirroring the US adult population was matched to opt-in panelists on demographic and consumer characteristics, and the resulting data were weighted to population benchmarks including age, sex, race or ethnicity, and education [62]. The exact matching and weighting specifications were determined by YouGov. Survey weights supplied by YouGov were applied in all analyses. Participants (N=1000) ranged in age from 18 to 91 years (mean 48.44, SD 17.76). The sample was approximately balanced by sex, with 48.6% (n=486) identifying as male, and 37.4% (n=374) reporting a high school education or less. The racial and ethnic composition was predominantly White (637/1000, 63.7%), followed by Hispanic (176/1000, 17.6%) and Black (116/1000, 11.6%). Details about participant characteristics can be found in Table 1.

**Table 1.** Demographic characteristics, N=1000 (unweighted vs weighted).

Characteristic	Unweighted, n (%)	Weighted, n (%)
Sex		
Female	510 (51)	514 (51.4)
Male	490 (49)	486 (48.6)
Ethnicity		
Hispanic or Latino	151 (15.1)	176 (17.6)
Non-Hispanic	849 (84.9)	824 (82.4)
Race^a^		
White	675 (67.5)	637 (63.7)
Hispanic	105 (10.5)	123 (12.3)
Black	95 (9.5)	116 (11.6)
Asian	53 (5.3)	58 (5.8)
Two or more races	29 (2.9)	26 (2.6)
Other	25 (2.5)	23 (2.3)
Native American	12 (1.2)	11 (1.1)
Middle Eastern	6 (0.6)	7 (0.7)
Education^a^		
Did not complete high school	39 (3.9)	67 (6.7)
High school graduate	321 (32.1)	307 (30.7)
Some college	181 (18.1)	194 (19.4)
2-year degree	93 (9.3)	95 (9.5)
4-year degree	213 (21.3)	208 (20.8)
Postgraduate	153 (15.3)	128 (12.8)
Family income (US $)^a^		
<30,000	240 (24.0)	260 (26.0)
30,000-59,999	232 (23.2)	237 (23.7)
60,000-99,999	222 (22.2)	210 (21.0)
100,000-149,999	157 (15.7)	150 (15.0)
≥150,000	91 (9.1)	88 (8.8)
Prefer not to say	58 (5.8)	57 (5.7)

a Weighted frequencies were rounded to whole numbers using total-preserving rounding. Percentages were calculated from unrounded weighted frequencies and may not sum to 100% because of rounding.

### Measures

All scales used a 5-point Likert agreement scale unless otherwise noted.

#### Usage Intention (Outcome Variable)

Participants’ intention to use the chatbot was measured using 4 items assessing the likelihood of use across various future time points (eg, “...after the survey?” “...1 month from today?”; α=.95, mean 2.68, SD 1.22).

#### Descriptive Norm

The descriptive norm was measured using 3 items [63] assessing participants’ perceptions of whether others similar to them use health chatbots (eg, “Most people like me use health chatbots,” “People similar to me find health chatbots helpful”; α=.84, mean 3.44, SD 0.75).

#### Injunctive Norm

The injunctive norm was measured using 3 items [64] assessing perceptions of whether important others and authorities approve of chatbot use (eg, “People who are important to me think I should use health chatbots,” “Health professionals would recommend that I use health chatbots”; α=.82, mean 3.91, SD 0.58).

#### Perceived Susceptibility

Assessment of perceived susceptibility to cancer involved a 3-item scale (α=.89, mean 2.89, SD 0.97). These items were developed by modifying existing measures from Scherr et al [65] and Champion and Skinner [66] and included statements such as “It is likely that I will develop cancer at some point in my life” and “People like me are at risk for getting cancer.”

#### Perceived Severity

Three items measuring perceived severity were adapted from Scherr et al [65] and Champion and Skinner [66]. Items included “Cancer is a serious health concern,” “Cancer is a disease that poses significant risks to health,” and “Cancer is a severe disease” (α=.85, mean 4.53, SD 0.63).

#### Perceived Health Benefits

The perceived benefits measure consisted of 6 items (α=.89, mean 3.38, SD 0.84). These items, drawing from scales by Champion and Skinner [66] and Walrave et al [67], assessed anticipated advantages, such as helping users “better understand my hereditary cancer risk” and enabling them to “gain information about genetic testing options from the chatbot.”

#### Self-Efficacy

Five items measuring self-efficacy were adapted from Champion et al [68] and Walrave et al [67]. The items evaluated participants’ confidence in their ability to use the chatbot, including aspects such as having access to the necessary device and internet, possessing the knowledge to use the chatbot, and feeling confident navigating its features (α=.82, mean 3.96, SD 0.72).

#### Perceived Ease of Use

Perceived ease of use was measured with 6 items adapted from Davis [69]. The scale included statements like “Learning to use this chatbot would be easy for me,” and “I would find this chatbot easy to use” (α=.91, mean 3.45, SD 0.74).

#### Trust

Trust in human-computer interaction was measured using a 12-item scale [70]. The scale assesses users’ trust in technology-based interactions, including perceptions of risk, benevolence, competence, and overall dependence or reliance on the system. Items were adapted to the health chatbot context (α=.94, mean 2.95, SD 0.77).

#### Privacy Concern

Privacy concern was measured using 4 items [71,72] assessing worry about data privacy and misuse (eg, “I am concerned that my personal health information could be misused by this chatbot,” and “I worry about the privacy of my health data shared with this chatbot”). Higher scores indicate greater privacy concern (α=.95, mean 3.71, SD 0.98).

### Data Analysis

SPSS Statistics (version 30.0; IBM Corp) was used for all analyses. To address RQ1, a 2-way multivariate ANOVA was conducted to examine the main and interactive effects of norm message type and chatbot type on the set of continuous dependent variables (health motivation factors, technology acceptance factors, and usage intention). Post hoc comparisons using a Bonferroni correction were conducted for any significant main effect of norm message type to identify specific group differences.

To test H1 and H2, multiple linear regression analyses were used to examine the direct associations between health motivation and technology acceptance factors and usage intention. Tests of the a priori hypotheses (H1 and H2) and of the focal effects on usage intention are treated as confirmatory; univariate effects on the remaining dependent variables are exploratory.

A sensitivity analysis (G*Power; version 3.1; Heinrich-Heine-Universität Düsseldorf) confirmed the study was well powered. With a sample size of 1000, α=.05, and power=0.80, the regression model could detect effects as small as f²=0.016. The 4-group norm-message comparison could detect *f*=0.10, below Cohen thresholds for small effects. The study was therefore sensitive to small effects, indicating that nonsignificant results are unlikely to reflect insufficient power.

### Ethical Considerations

This study protocol was reviewed and approved by the Institutional Review Board (IRB) at the University of Utah (approval number: IRB_00186651). Informed consent was obtained from all participants at the beginning of the online survey. Only participants who agreed to the consent form were allowed to proceed to the survey questions.

## Results

### Effects of Message Features (RQ1)

**Figure 3.**
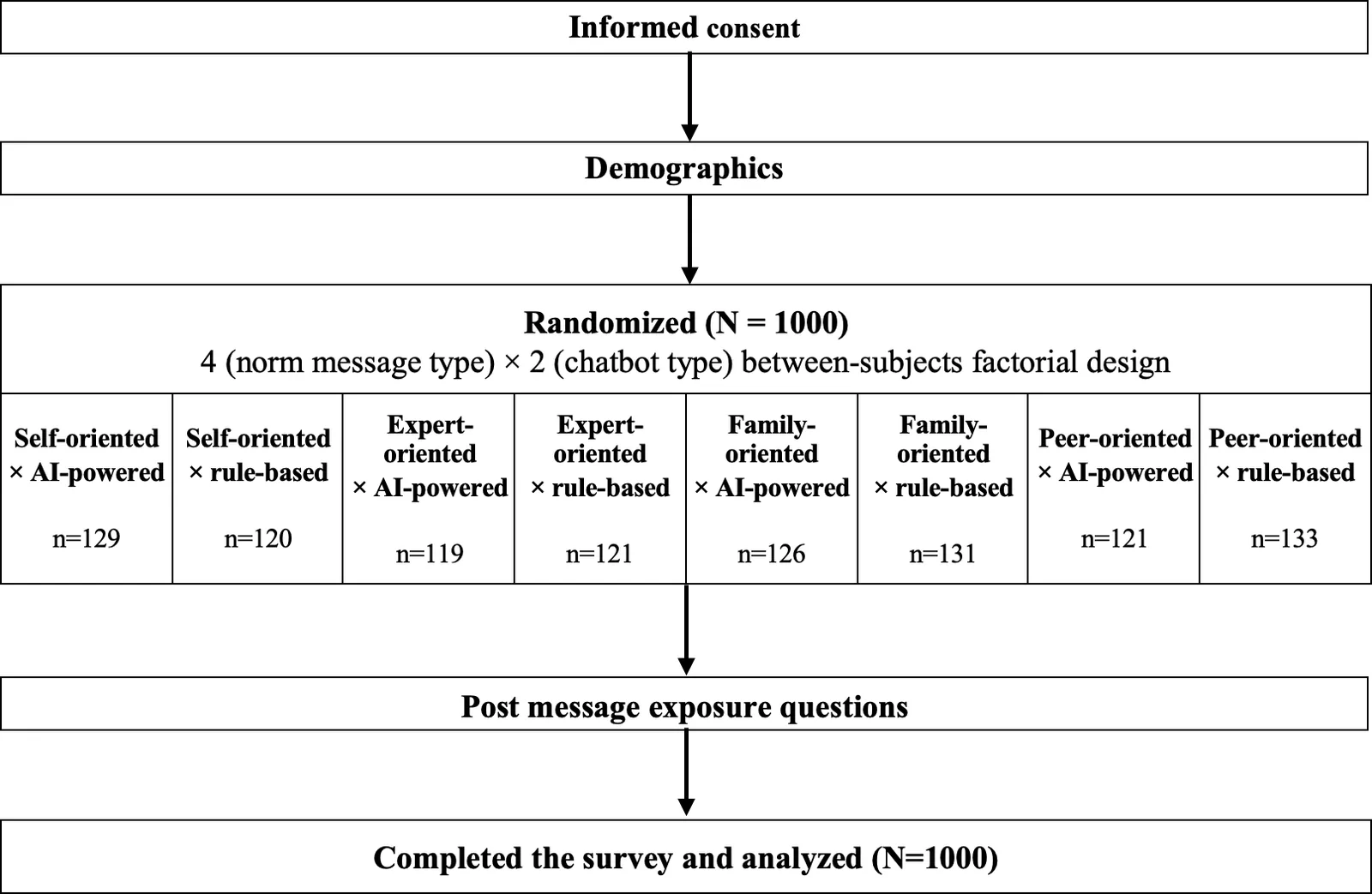
Participant flow diagram. The experiment was administered as a single-session, cross-sectional online survey. A target sample mirroring the US adult population was matched to opt-in panelists on demographic and consumer characteristics. The number of panelists invited, screened, or excluded prior to the delivery of the final matched sample was determined by the survey vendor (YouGov) and was not available to the investigators.

The multivariate tests indicated statistically significant effects for both independent variables and their interaction on the combined set of dependent variables (Figure 3). The main effect of the social norm message was significant, Pillai trace=0.103; *F*_30,3156_=3.73; *P*<.001; partial η²=0.034. The main effect of chatbot type was also significant, Pillai trace=0.028; *F*_10,1050_=3.02; *P*<.001; partial η²=0.028. Furthermore, a significant multivariate interaction effect was observed, Pillai trace=0.109; *F*_30,3156_=3.98; *P*<.001; partial η²=0.036. Tables 2 and 3 report means and SD by condition.

**Table 2.** Main effects of norm messages on outcomes^a^.

Characteristics	Self-oriented, mean (SD)	Expert-oriented, mean (SD)	Family-oriented, mean (SD)	Peer-oriented, mean (SD)	*F* test (*df*)
Descriptive norm	3.44 (0.74)	3.39 (0.76)	3.44 (0.70)	3.63 (0.77)	5.17^b^ (3, 1059)
Injunctive norm	3.90 (0.57)	3.90 (0.58)	3.91 (0.55)	4.06 (0.59)	5.05^b^ (3, 996)
Perceived health benefit	3.49 (0.79)	3.39 (0.81)	3.43 (0.77)	3.46 (0.83)	0.72 (3, 996)
Perceived susceptibility	2.86 (1.00)	2.90 (1.02)	2.85 (0.97)	3.03 (1.05)	1.75 (3, 996)
Perceived severity	4.56 (0.62)	4.46 (0.61)	4.57 (0.58)	4.63 (0.64)	3.51^c^ (3, 996)
Ease of use	3.52 (0.71)	3.42 (0.73)	3.46 (0.68)	3.48 (0.74)	0.86 (3, 996)
Self-efficacy	3.90 (0.71)	3.92 (0.73)	3.91 (0.68)	4.14 (0.74)	6.83^d^ (3, 996)
Trust	2.94 (0.76)	2.95 (0.78)	3.06 (0.72)	3.14 (0.78)	4.08^b^ (3, 996)
Privacy concern	3.93 (1.00)	3.74 (1.02)	3.51 (0.95)	3.35 (1.03)	16.76^d^ (3, 996)
Usage intention	2.69 (1.20)	2.61 (1.21)	2.91 (1.15)	2.92 (1.24)	4.45^b^ (3, 996)
N after weight	262	261	277	267	—^e^

a Outcomes were measured using 5-point Likert scales (1 to 5).

b *P*<.01.

c *P*<.05.

d *P*<.001.

e Not applicable.

**Table 3.** Main effects of chatbot type on outcomes^a^.

Characteristics	AI, mean (SD)	Rules-based, mean (SD)	*F* test (*df*)
Descriptive norm	3.52 (0.74)	3.43 (0.76)	4.25 (1, 1059)^b^
Injunctive norm	3.94 (0.56)	3.94 (0.57)	0.00 (1, 1059)
Perceived health benefit	3.48 (0.79)	3.40 (0.80)	3.02 (1, 1059)
Perceived susceptibility	2.80 (1.00)	3.03 (1.01)	13.51^c^ (1, 1059)
Perceived severity	4.53 (0.60)	4.58 (0.62)	1.82 (1, 1059)
Ease of use	3.50 (0.70)	3.43 (0.73)	2.69 (1, 1059)
Self-efficacy	3.97 (0.70)	3.97 (0.73)	0.00 (1, 1059)
Trust	3.08 (0.74)	2.96 (0.76)	7.02^d^ (1, 1059)
Privacy concern	3.57 (1.00)	3.69 (1.01)	3.84 (1, 1059)
Usage intention	2.87 (1.19)	2.69 (1.22)	5.90^b^ (1, 1059)
N after weight	541	526	—^e^

a Outcomes were measured using 5-point Likert scales (1 to 5).

b *P*<.05.

c *P*<.001.

d *P*<.01.

e Not applicable.

### Main Effects of Social Norm Messages

**Figure 4.**
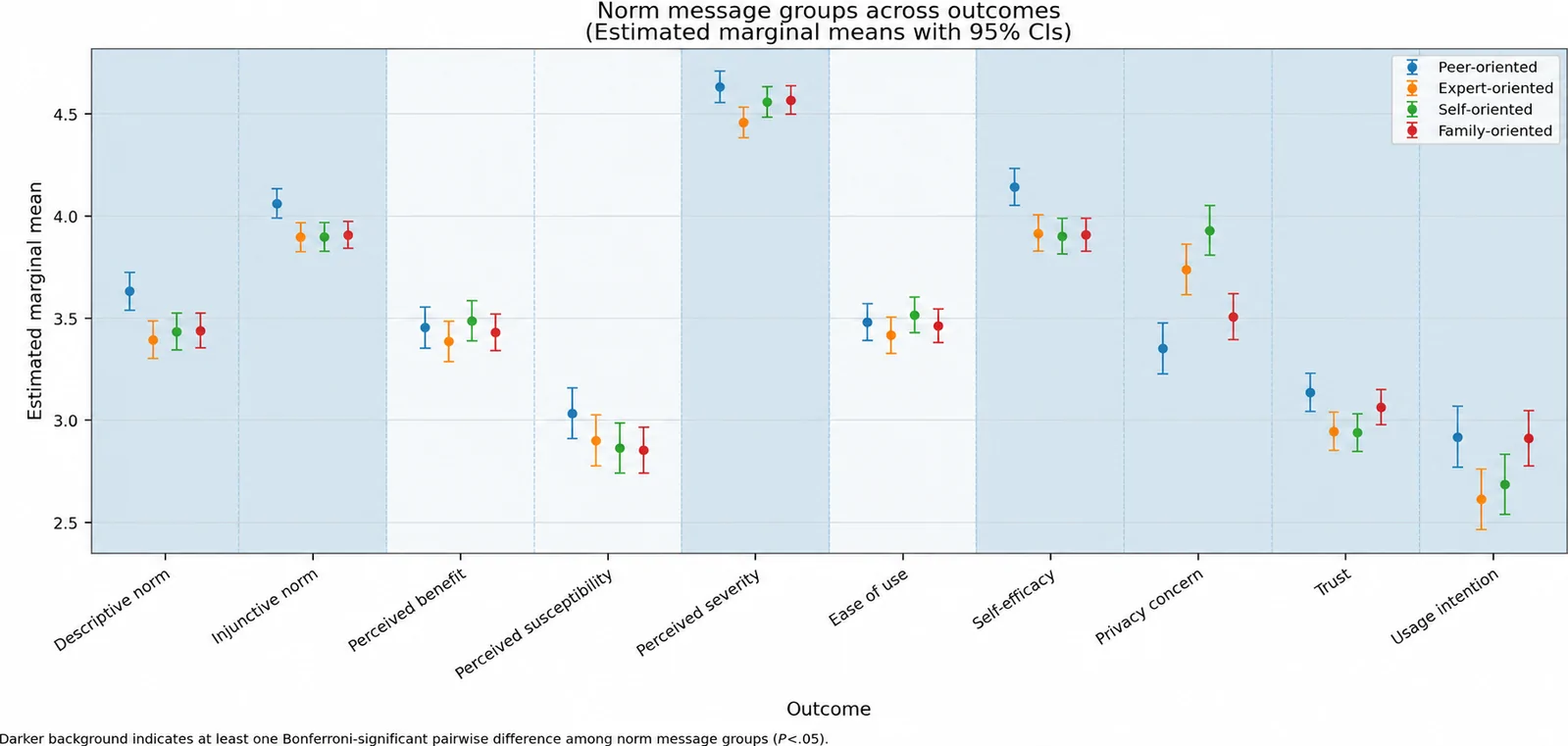
Norm message groups across outcomes (estimated marginal means with 95% CIs).

The main effect of norm messages (Figure 4) was statistically significant for usage intention (*F*_3,996_=4.45; *P*=.004), descriptive norm (*F*_3,1059_=5.17; *P*=.002), injunctive norm (*F*_3,996_=5.05; *P*=.002), perceived severity (*F*_3,996_=3.51; *P*=.02), self-efficacy (*F*_3,996_=6.83; *P*<.001), trust (*F*_3,996_=4.08; *P*=.007), and privacy concern (*F*_3,996_=16.76; *P*<.001). The main effect of norm messages was not significant for perceived susceptibility (*P*=.15), perceived benefit (*P*=.54), or ease of use (*P*=.46).

Bonferroni posthoc comparisons revealed distinct patterns across the 4 norm message framing conditions (Figure 4 and Multimedia Appendix 1). For usage intention, the peer-oriented message group (mean 2.92, SD 1.24) was significantly higher than the expert-oriented message group (mean 2.61, SD 1.21, mean difference [MD]=0.31; *P*=.02). The family-oriented message group (mean 2.91, SD 1.15) was also significantly higher than the expert-oriented message group (MD=0.33; *P*=.006) and the self-oriented message group (mean 2.69, SD 1.20, MD=0.27; *P*=.04).

For the descriptive norm, the peer-oriented message group (mean 3.63, SD 0.77) had significantly higher scores than the expert-oriented message group (mean 3.39, SD 0.76, MD=0.24; *P*=.002) and the self-oriented message group (mean 3.44, SD 0.74, MD=0.19; *P*=.03). For the injunctive norm, the peer-oriented message group (mean 4.06, SD 0.59) scored significantly higher than the expert-oriented message group (mean 3.90, SD 0.58, MD=0.17; *P*=.005), the self-oriented message group (mean 3.90, SD 0.57, MD=0.16; *P*=.007), and the family-oriented message group (mean 3.91, SD 0.55, MD=0.15; *P*=.02).

For perceived severity, the peer-oriented message group (mean 4.63, SD 0.64) had significantly higher scores than the expert-oriented message group (mean 4.46, SD 0.61, MD=0.18; *P*=.007). For self-efficacy, the peer-oriented message group (mean 4.14, SD 0.74) had significantly higher scores than the expert-oriented message group (mean 3.92, SD 0.73, MD=0.23; *P*=.002), the self-oriented message group (mean 3.90, SD 0.71, MD=0.25; *P*<.001), and the family-oriented message group (mean 3.91, SD 0.68, MD=0.22; *P*=.002).

For trust, the peer-oriented message group (mean 3.14, SD 0.78) was significantly higher than the expert-oriented message group (mean 2.95, SD 0.78, MD=0.19; *P*=.03) and the self-oriented message group (mean 2.94, SD 0.76, MD=0.19; *P*=.02).

For privacy concerns, the self-oriented message group (mean 3.93, SD 1.00) was significantly higher than the peer-oriented message group (mean 3.35, SD 1.03, MD=0.59; *P*<.001) and the family-oriented message group (mean 3.51, SD 0.95, MD=0.45; *P*<.001). The expert-oriented message group (mean 3.74, SD 1.02) was also significantly higher than the peer-oriented message group (MD=0.40*; P*<.001) and the family-oriented message group (MD=0.26; *P*=.02).

### Main Effects of Chatbot Type

**Figure 5.**
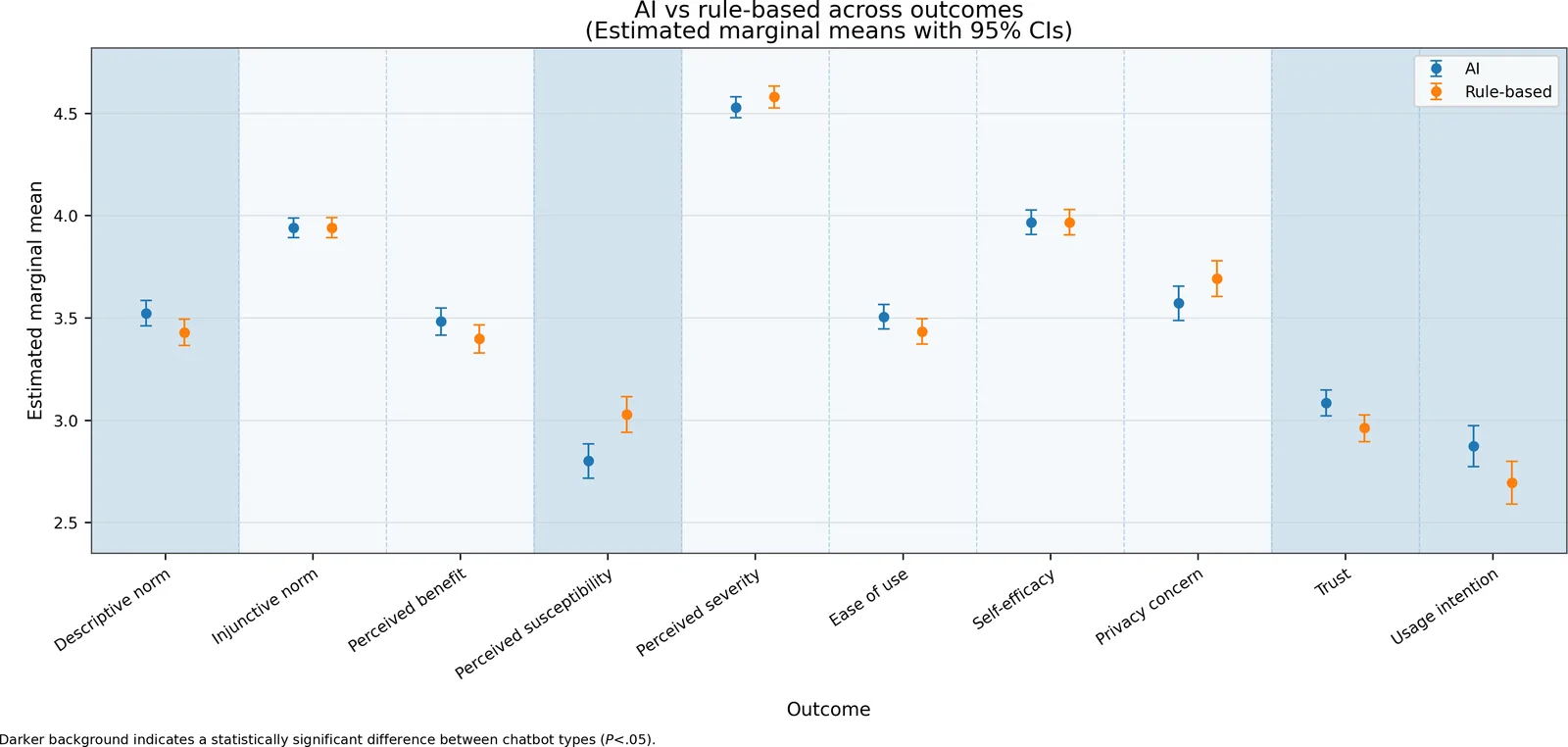
AI vs rule-based chatbot types across outcomes (estimated marginal means with 95% CIs).

The main effect of chatbot type (AI vs rule-based) was statistically significant for 4 variables (Figure 5). Participants assigned to the AI condition reported significantly stronger usage intention, *F*_1,1059_=5.90, *P*=.02, a higher descriptive norm*, F*_1,1059_=4.25, *P*=.04, lower perceived susceptibility*, F*_1,1059_=13.51; *P*<.001), and greater trust in the human-computer interface *F*_1,1059_=7.02; *P*=.008). The main effect of type was not significant for injunctive norm (*P*>.99), perceived severity (*P*=.18), perceived benefit (*P*=.08), self-efficacy (*P*>.99), or ease of use (*P*=.10). The difference in privacy concern approached statistical significance (*F*_1,1059_=3.84; *P*=.05), with the rule-based condition showing slightly higher concern (mean 3.69, SD 1.01; 95% CI 3.61-3.78) than the AI condition (mean 3.57, SD 1.00; 95% CI 3.49-3.66). However, the 95% CIs overlapped, indicating uncertainty about the magnitude and reliability of this difference.

### Interaction Effects

The interaction effect between norm messages and chatbot type was statistically significant for usage intention (*F*_3,1059_=8.41; *P*<.001), descriptive norm (*F*_3,1059_=8.63; *P*<.001), injunctive norm (*F*_3,1059_=6.32; *P*<.001), perceived benefit (*F*_3,1059_=5.29; *P*=.001), self-efficacy (*F*_3,1059_=6.83; *P*<.001), and privacy concern (*F*_3,1059_=6.93; *P*<.001). The interaction was not significant for perceived susceptibility (*P*=.23), perceived severity (*P*=.06), ease of use (*P*=.07), or trust (*P*=.14).

With the family-oriented norm message, the AI chatbot type scored significantly higher on usage intention (MD=0.67; *P*<.01), descriptive norm (MD=0.43; *P*<.01), injunctive norm (MD=0.22; *P*<.01), perceived benefit (MD=0.33; *P*<.01), ease of use (MD=0.21; *P*=.01), self-efficacy (MD=0.32; *P*<.01), and trust (MD=0.27; *P*=.002). In contrast, the rule-based chatbot type scored significantly higher on privacy concern (MD=−0.45; *P*<.01).

With the expert-oriented norm message, the rule-based chatbot type scored significantly higher on usage intention (MD=−0.38; *P*=.01), descriptive norm (MD=−0.20; *P*=.03), injunctive norm (MD=−0.18; *P*=.01), perceived severity (MD=−0.15; *P*=.04), and privacy concern (MD=−0.28; *P*=.03).

Results were mixed within the peer-oriented norm message condition. The AI chatbot type had significantly higher perceived benefit (MD=0.21; *P*=.04) and privacy concern (MD=0.28; *P*=.03), while the rule-based chatbot type scored higher on perceived susceptibility (MD=−0.38; *P*=.003).

For the self-oriented norm message, only the rule-based chatbot type scored significantly higher on perceived susceptibility (MD=−0.28; *P*=.02). All other comparisons were nonsignificant.

### Regression Analyses (H1 and H2)

Multicollinearity diagnostics were acceptable. The highest variance inflation factor was 3.61 for trust, below the conventional threshold of 5. The overall model was statistically significant, *F*_9,990_=113.41*; P*<.001, explaining 50.8% of the variance in usage intention (*R*=0.712, *R*²=0.508, Adjusted *R*²=0.503). H1 predicted that health motivation factors would have a positive association with usage intention and received partial support. Four of the 5 factors operated as predicted: descriptive norm (β=0.087; *P*=.003), injunctive norm (β=0.078; *P*=.009), perceived susceptibility (β=0.051; *P*=.03), and perceived benefits (β=0.253; *P*<.001) all demonstrated significant positive associations with usage intention. However, the hypothesis was not supported for perceived severity, which showed a significant negative association (β=−0.047; *P*=.049).

As H2 predicted, trust had a positive association with usage intention (β=0.33; *P*<.001), and privacy concern had a negative association (β=−0.11; *P*<.001). However, ease of use (*P*=.09) and self-efficacy (*P*=.08) were not statistically significant predictors.

To verify that these associations were not attributable to demographic differences, we reestimated the model with demographic controls (age, sex, race, and education) entered as a prior block. Demographics alone explained little of the variance in usage intention (*R*²=0.017), and the HTAM coefficients remained substantively unchanged when demographics were included; only race or ethnicity was an independent predictor in the full model. Full results are reported in Multimedia Appendix 2.

## Discussion

### Principal Findings

This study advances research on digital health adoption by proposing and empirically testing HTAM, an integrative framework that bridges technology acceptance and health behavior perspectives. Consistent with prior critiques of fragmented technology adoption models [16,18], the findings demonstrate that both normative message framing and chatbot type significantly shape users’ motivational and acceptance-related beliefs.

### Theoretical Implications

The findings provide support for HTAM as a complementary framework to existing adoption models. Traditional TAM emphasizes cognitive evaluations such as ease of use and usefulness, while health behavior theories focus on perceived threats and benefits. By integrating these perspectives, HTAM captures the dual nature of health technologies as both technical systems and health management tools. The present results show that variables from both pathways simultaneously contributed to usage intention, accounting for over half of its variance.

Notably, perceived benefits emerged as the strongest motivational predictor, underscoring the importance of framing chatbots as effective tools for improving health outcomes. At the same time, trust and privacy concerns played central roles in shaping adoption, highlighting the limitations of usability-focused models in health contexts. These findings reinforce prior calls [23,32] to expand technology acceptance frameworks to include ethical and relational dimensions, particularly when technologies handle sensitive personal information.

The nonsignificant effects of ease of use and self-efficacy in the regression model suggest that basic usability may function as a threshold condition rather than a primary driver of intention. One explanation is that contemporary users bring a relatively high baseline of digital literacy and routine experience with conversational interfaces such as messaging apps and virtual assistants; when a system is presumed easy to operate, ease of use loses discriminating power, and motivational and relational factors, perceived benefits, trust, and privacy concerns, become the more decisive determinants of intention. Another plausible explanation for these results involves the methodological design of the study. Because participants evaluated the chatbots based on written descriptions and static stimuli rather than direct, hands-on interaction, their assessments of usability and personal capability were necessarily hypothetical.

The association between perceived severity and usage intention was negative. Although health behavior theories typically predict positive effects of perceived severity on protective behavior [25,73], fear-appeal research shows that this relationship is conditional: threat motivates protective action when paired with sufficient efficacy, but can trigger avoidance or disengagement when efficacy is low [74]. Because participants encountered cancer-related information without a strong sense that using a chatbot would reduce their risk, heightened severity may have prompted avoidance rather than engagement. This implies that cancer prevention chatbots emphasizing disease seriousness without conveying actionable efficacy may inadvertently depress use. We interpret this effect cautiously, however, as the coefficient was small and marginally significant (β=−0.047; *P*=.049), warranting replication.

### Effects of Normative Messaging

Normative message framing played a substantial role in shaping users’ beliefs and intentions. Peer-oriented messages consistently strengthened descriptive norms, injunctive norms, self-efficacy, trust, and usage intention. This pattern aligns with social norms theory, which emphasizes the persuasive power of perceived similarity and social proximity [36,37,44]. When individuals perceive that “people like me” engage in a behavior, they are more likely to view that behavior as appropriate and attainable.

Family-oriented messages also increased usage intention, reflecting the motivational influence of relational responsibility and social connectedness. In contrast, expert-oriented norms were generally less effective, challenging assumptions that professional authority alone is sufficient to motivate digital health engagement. In chatbot-mediated environments, institutional endorsements may appear distant or impersonal, reducing their persuasive impact.

Normative messages also shaped privacy concerns and trust. Self-oriented and expert-oriented messages were associated with higher privacy concerns, whereas peer-oriented and family-oriented frames were associated with lower privacy concerns. One interpretation is that social embeddedness normalizes data sharing and mitigates perceived risk. Alternatively, self-oriented and expert-oriented messages may have drawn attention to the information transaction itself, prompting closer scrutiny of data use, whereas peer-oriented and family-oriented framing directed attention away from privacy. These accounts differ ethically: if social framing merely diverts attention from legitimate concerns rather than resolving them, recommending it to reduce privacy concerns would be problematic. Designers should therefore pair social framing with transparent data practices and future research should disentangle these mechanisms.

### Effects of Chatbot Type

Chatbot type exerted selective effects on user perceptions. Participants in the AI-powered condition reported higher trust and usage intention. This distinction matters because the way a chatbot is described, before any interaction occurs, can itself shape whether individuals choose to engage with it. Although prior literature often suggests that AI systems may elicit lower trust due to algorithmic opacity, uncertainty about data processing, and concerns about limited human oversight [58,60], the present findings indicate a more nuanced dynamic. In a message experiment, participants evaluated described capabilities rather than interacting with a functioning system; therefore, the promised benefits of an AI-powered chatbot may have outweighed concerns about opacity or error. Whether this positive effect persists after users encounter the chatbot’s actual behavior, including inaccurate responses, uncertainty, or limitations in contextual understanding, remains an important question for future research. Another possible explanation is that conversational flexibility and personalization enhance perceived competence and responsiveness, which are central components of trust formation in human-computer interaction [9,56,59]. When a system appears capable of understanding user input and providing tailored feedback, users may interpret this adaptability as expertise and relational attentiveness. Additionally, the novelty of AI-powered systems may have positively biased user evaluations, as emerging technologies often evoke curiosity and heightened expectations.

The marginal difference in privacy concerns reflects ongoing ambivalence toward AI systems. Although participants generally trusted AI chatbots more than rule-based chatbots, uncertainty about data processing remains salient [50,58]. This tension underscores the importance of transparency and explainability in health technology design that relied on AI.

For practitioners and developers, the results suggest several actionable strategies. Incorporating peer-based social cues can enhance engagement. Emphasizing concrete health benefits may strengthen motivation and trust. Designers should also calibrate risk communication to avoid inducing excessive fear that may discourage use. Health care organizations implementing chatbots should consider audience segmentation and message tailoring, as responses to normative and technological cues may vary across population subgroups.

Practically, because privacy concerns can suppress usage intention even when AI chatbots increase trust and perceived usefulness, reducing data-related apprehension is essential for adoption. Evidence from recent health-chatbot and LLM privacy literature suggests a layered approach: collect only task-relevant data, sanitize or deidentify sensitive inputs locally where feasible, obtain clear upfront consent, explain in plain language what data are stored and for how long, and provide simple controls to delete data or opt out of model training [75-78].

### Limitations

Several limitations warrant consideration. First, the study relied on self-reported intention rather than observed behavior, a common limitation in technology adoption research. Second, participants evaluated described chatbot features rather than engaging in live interaction, limiting ecological validity. Third, the focus on cancer-related information may restrict generalizability, as cognitive response intensity varies across health domains. Fourth, the sample was predominantly White (637/1000, 63.7%) and drawn entirely from US adults, a population situated within a broadly Western, individualistic cultural context. Because the persuasive force of normative appeals depends on which social referents are most salient and identity-relevant, the relative effectiveness of the peer-oriented and family-oriented frames observed here may not transfer completely to other cultural settings. Fifth, although HTAM conceptually positions the technology acceptance and health motivation constructs between design features and usage intention, the present study tests these component paths separately rather than estimating formal indirect effects; mediation or structural equation analyses would be needed to evaluate the framework’s full pathway structure, and we encourage such tests in future research. Sixth, the study did not include manipulation-check items verifying that participants correctly identified the assigned norm source and chatbot type. Although the predicted effects of the norm manipulation on normative and related constructs provide indirect evidence that it registered, future research should incorporate explicit checks. Seventh, the chatbot-type manipulation combined system architecture with the personalization and authorship of the information: the AI-powered condition was described as learning and personalizing, whereas the rule-based condition was described as scripted by a health care team for consistency. The observed effects of chatbot type therefore reflect this composite of features rather than architecture alone. Future work could disentangle these dimensions. Eighth, this study did not systematically examine demographic moderation effects. Prior research suggests that factors such as age, digital literacy, and health status may influence responses to both AI systems and health messaging. Future research should explicitly test these moderating effects to better understand heterogeneity in user responses.

Future research should also extend HTAM by examining its applicability across varied health contexts (eg, mental health, chronic disease management, and preventive care) as well as across different technological modalities, including AI diagnostic systems, wearable devices, and mobile health applications. Mental health support warrants particular attention. A growing body of work on AI chatbots for mental health points to trust and privacy dynamics that differ from those in preventive contexts such as cancer screening: disclosures are more sensitive, the consequences of inaccurate or unsafe responses are more acute, and concerns about data handling and the limits of automated support are more pronounced [49,58,60]. Examining how the technology acceptance and health motivation pathways in HTAM operate under these heightened stakes would test the framework’s boundaries and inform the responsible design of mental health chatbots. Cross-cultural and cross-national studies could clarify whether the differential efficacy of normative messaging is contingent on cultural values such as individualism-collectivism, power distance, and uncertainty avoidance. Such work would help establish the boundary conditions of HTAM and indicate whether normative message tailoring should be culturally adapted rather than applied uniformly across populations. Future studies may also incorporate additional external variables, such as transparency cues, human oversight, or adaptive personalization, and adopt longitudinal studies to clarify causal pathways.

### Conclusions

This study proposed and tested the HTAM to explain how social and technological design features jointly influence engagement with health chatbots. By integrating technology acceptance and health motivation perspectives, HTAM provides a comprehensive framework for understanding users’ adoption intentions. The findings demonstrate that normative message framing and chatbot type significantly shape perceived benefits, trust, social norms, and privacy concerns, which in turn predict usage intention. Peer-oriented and family-oriented messages were particularly effective, especially when paired with AI-powered systems. In contrast, ease of use and self-efficacy played a limited role in this experimental context. Overall, this study highlights the importance of designing health chatbots that balance functionality, social influence, and ethical considerations to promote sustained and meaningful engagement.

## Supplementary material

10.2196/97773 Multimedia Appendix 1 Pairwise comparisons of social norm messages.

10.2196/97773 Multimedia Appendix 2 Hierarchical regression predicting usage intention with demographic controls.

10.2196/97773 Checklist 1 CONSORT-EHEALTH checklist (V1.6.2).

## Abbreviations

CONSORT-EHEALTH: Consolidated Standards of Reporting Trials of Electronic and Mobile Health Applications and Online Telehealth
HTAM: health technology acceptance model
IRB: Institutional Review Board
LLM: large language model
MD: mean difference
OSF: Open Science Framework
RQ: research question
TAM: technology acceptance model
