# Supplementary material for: The Impact of Chatbot Type and Normative Messaging on Chatbot Usage Intention Based on the Health Technology Acceptance Model: Randomized Controlled Trial
Source: J Med Internet Res. 2026 Sep 15;28:e97773. doi: 10.2196/97773 (PMC13577661; doi:10.2196/97773)
Supplement: Supplementary file 1 [file jmir-v28-e97773-s003.pdf]

CONSORT-EHEALTH Checklist V1.6.2 Report	Manuscript Number	97773
**(based on CONSORT-EHEALTH V1.6), available at [http://tinyurl.com/consort-ehealth-v1-6].**		
**Date completed**		
8/17/2026 23:43:22		
**by**		
Yi Liao		
TITLE		
**1a-i) Identify the mode of delivery in the title**		
**1a-ii) Non-web-based components or important co-interventions in title**		
The Impact of Chatbot Type and Normative Messaging on Chatbot Usage Intention: Randomized Controlled Trial Based on the Health Technology Acceptance Model". The mode of delivery is identified in the title by "Chatbot", and the chatbot type contrast (AI-powered vs rule-based) is the focus of the study. The web-based nature of the trial is stated in the first line of the Abstract Methods: "we conducted a 4×2 between-subjects online experiment with 1000 US adults recruited from a nationally representative YouGov panel."		
**1a-iii) Primary condition or target group in the title**		
Not applicable. There were no non-web-based components and no co-interventions.		
ABSTRACT		
**1b-i) Key features/functionalities/components of the intervention and comparator in the METHODS section of the ABSTRACT**		
**1b-ii) Level of human involvement in the METHODS section of the ABSTRACT**		
Participants were randomized to 1 of 8 conditions varying norm message type (self-, peer-, expert-, or family-oriented) and chatbot type (artificial intelligence [AI] powered or rule-based) in a cancer prevention and genetic risk information scenario." The abstract also names the guiding theory: "This study proposed and tested the health technology acceptance model (HTAM)."		
**1b-iii) Open vs. closed, web-based (self-assessment) vs. face-to-face assessments in the METHODS section of the ABSTRACT**		
**1b-iv) RESULTS section in abstract must contain use data**		
**1b-v) CONCLUSIONS/DISCUSSION in abstract for negative trials**		
INTRODUCTION		
**2a-i) Problem and the type of system/solution**		
**2a-ii) Scientific background, rationale: What is known about the (type of) system**		
The Introduction defines the problem and the class of system: "health chatbots, also known as health conversational agents, have gained significant traction, evolving from simple administrative tools to sophisticated agents capable of delivering personalized medical information, symptom assessment, and behavioral support," yet "Although health chatbots are widely deployed, users' willingness to adopt and engage with these systems varies substantially." The system of interest is a stand-alone, consumer-facing preventive health chatbot delivering cancer prevention and hereditary risk information, intended to complement rather than replace clinical care; the goal of the study is to explain usage intention, "the immediate psychological precursor to actual adoption."		
METHODS		
**3a) CONSORT: Description of trial design (such as parallel, factorial) including allocation ratio**		
**3b) CONSORT: Important changes to methods after trial commencement (such as eligibility criteria), with reasons**		
RQ1: What is the relationship between design features (norm message type, chatbot type) and health motivation factors, technology acceptance factors, and usage intention? H1: Health motivation factors (descriptive norm, injunctive norm, perceived susceptibility, perceived severity, perceived benefits) will be positively associated with usage intention. H2: Technology acceptance factors (ease of use, self-efficacy, trust) will be positively associated with usage intention, while privacy concerns will be negatively associated with usage intention.		
**3b-i) Bug fixes, Downtimes, Content Changes**		
**4a) CONSORT: Eligibility criteria for participants**		
This study employed a 4 × 2 between-subjects factorial experimental design with two independent variables: norm message type (self-, peer-, expert-, family-oriented) and chatbot type (AI-powered vs. rules-based). Participants were randomly assigned to one of eight experimental conditions." Allocation was equal (1:1:1:1:1:1:1:1) across the eight cells; realized cell sizes ranged from n=119 to n=133 (Figure 3).		
**4a-i) Computer / Internet literacy**		
**4a-ii) Open vs. closed, web-based vs. face-to-face assessments:**		
**4a-iii) Information giving during recruitment**		
This was a closed, purely web-based study with no face-to-face contact between the study team and participants: "Representativeness was achieved through YouGov's standard sample-matching and weighting procedure: a target sample mirroring the U.S. adult population is matched to opt-in panelists on demographic and consumer characteristics." Participants were quasi-anonymous to the investigators, who received a de-identified dataset. Duplicate or multiple identities were prevented by the vendor's authenticated single-invitation panel infrastructure; each panelist could complete the survey once.		
**4b) CONSORT: Settings and locations where the data were collected**		
There were no changes to the methods after the study commenced. All data were collected in a single fielding period in October 2025, and the analysis plan distinguishing confirmatory from exploratory tests was applied as specified: "Tests of the a priori hypotheses (H1, H2) and of the focal effects on usage intention are treated as confirmatory; univariate effects on the remaining dependent variables are exploratory."		
**4b-i) Report if outcomes were (self-)assessed through online questionnaires**		
**4b-ii) Report how institutional affiliations are displayed**		
All outcomes were self-reported through online questionnaires administered immediately after stimulus exposure: "Following exposure to the assigned stimulus, participants completed measures assessing health motivation factors, technology acceptance factors, and intention to use the chatbot based on the provided scenarios," and "All scales used a 5-point Likert agreement scale unless otherwise noted." No objective or behavioral outcomes were collected.		
**5) CONSORT: Describe the interventions for each group with sufficient details to allow replication, including how and when they were actually administered**		
**5-i) Mention names, credential, affiliations of the developers, sponsors, and owners**		
**5-ii) Describe the history/development process**		
**5-iii) Revisions and updating**		
**5-iv) Quality assurance methods**		
**5-v) Ensure replicability by publishing the source code, and/or providing screenshots/screen-capture video, and/or providing flowcharts of the algorithms used**		

5-vi) Digital preservation

5-vii) Access

5-viii) Mode of delivery, features/functionalities/components of the intervention and comparator, and the theoretical framework

Not applicable in the conventional sense: the chatbot did not exist as a product that an end user could access.

5-ix) Describe use parameters

The health content in the stimuli concerned general cancer prevention and hereditary risk information and was drafted and reviewed by the research team for accuracy before fielding. Outcome measurement quality was addressed through previously validated scales with high internal consistency (Cronbach alpha = .82 to .95, reported for each measure in the Measures section).

5-x) Clarify the level of human involvement

5-xi) Report any prompts/reminders used

5-xii) Describe any co-interventions (incl. training/support)

No prompts or reminders of any kind were used. The study was a single-session, cross-sectional online survey with no follow-up contact, no email or SMS prompts, and no re-engagement attempts.

6a) CONSORT: Completely defined pre-specified primary and secondary outcome measures, including how and when they were assessed

Participants were US adults aged 18 years or older recruited from the YouGov opt-in panel: "Participants were recruited in October 2025 from a nationally representative sample using the YouGov panel," and "Participants (N = 1,000) ranged in age from 18 to 91 years (M = 48.44, SD = 17.76)." No clinical eligibility criteria were applied, since the study assessed responses to described health technology rather than delivering care. Panel membership and completion of an English-language web survey were de facto criteria.

6a-i) Online questionnaires: describe if they were validated for online use and apply CHERRIES items to describe how the questionnaires were designed/deployed

6a-ii) Describe whether and how “use” (including intensity of use/dosage) was defined/measured/monitored

6a-iii) Describe whether, how, and when qualitative feedback from participants was obtained

6b) CONSORT: Any changes to trial outcomes after the trial commenced, with reasons

All data were collected online across the United States in October 2025 through the YouGov panel platform. Participants completed the study remotely, at a time and place of their choosing, on their own internet-connected devices. There were no clinical or laboratory settings involved.

7a) CONSORT: How sample size was determined

7a-i) Describe whether and how expected attrition was taken into account when calculating the sample size

7b) CONSORT: When applicable, explanation of any interim analyses and stopping guidelines

All outcomes were assessed once, immediately after stimulus exposure, on 5-point Likert agreement scales. Primary outcome: "Usage Intention (Outcome Variable). Participants' intention to use the chatbot was measured with four items assessing the likelihood of use across various future time points (e.g., '... after the survey?', '...one month from today?') (alpha = .95, M = 2.68, SD = 1.22)." Secondary outcomes and their sources are defined in the Measures section: descriptive norm (3 items, alpha = .84), injunctive norm (3 items, alpha = .82), perceived susceptibility (3 items, alpha = .89), perceived severity (3 items, alpha = .85), perceived health benefits (6 items, alpha = .89), self-efficacy (5 items, alpha = .82), perceived ease of use (6 items, alpha = .91), trust (12 items, alpha = .94), and privacy concern (4 items, alpha = .95).

8a) CONSORT: Method used to generate the random allocation sequence

No outcomes were added, dropped, or redefined after data collection began. The confirmatory-versus-exploratory division was specified in the analysis plan and reported as such: "Tests of the a priori hypotheses (H1, H2) and of the focal effects on usage intention are treated as confirmatory; univariate effects on the remaining dependent variables are exploratory."

8b) CONSORT: Type of randomisation; details of any restriction (such as blocking and block size)

Not applicable. No interim analyses were conducted and no stopping rules were defined. All data were collected in a single fielding wave in October 2025 and analyzed once, after the full sample of N=1000 was delivered.

9) CONSORT: Mechanism used to implement the random allocation sequence (such as sequentially numbered containers), describing any steps taken to conceal the sequence until interventions were assigned

Random assignment to one of the eight conditions was performed automatically by the survey platform's built-in randomization function at the point of survey entry, after informed consent and the demographic block. Assignment was computer-generated with equal probability across the eight cells; no investigator was involved in generating or applying the sequence. Realized cell sizes ranged from n=119 to n=133 (Figure 3), consistent with unrestricted random allocation.

10) CONSORT: Who generated the random allocation sequence, who enrolled participants, and who assigned participants to interventions

Simple (unrestricted) randomization with equal allocation across the eight conditions of the 4 x 2 factorial design. No blocking, stratification, or minimization was used. The resulting distribution is reported in Figure 3: self-oriented n=249, expert-oriented n=240, family-oriented n=257, peer-oriented n=254; AI-powered n=495, rule-based n=505.

11a) CONSORT: Blinding - If done, who was blinded after assignment to interventions (for example, participants, care providers, those assessing outcomes) and how

11a-i) Specify who was blinded, and who wasn’t

11a-ii) Discuss e.g., whether participants knew which intervention was the “intervention of interest” and which one was the “comparator”

Participants were blind to their assigned condition: each saw only one of the eight stimuli and was not informed that alternative norm framings or chatbot-type descriptions existed, so participants could not know which condition was the control. Because the design is single-exposure and between-subjects, participants could not compare conditions. There were no outcome assessors or care providers to blind, since all outcomes were self-reported and delivery was fully automated. The investigators were not blinded during analysis, as condition membership was coded in the delivered dataset; however, all outcomes were machine-scored self-report scales, limiting the scope for assessor bias.

11b) CONSORT: If relevant, description of the similarity of interventions

Allocation was implemented automatically by the survey software immediately after consent and before stimulus presentation. Because assignment was machine-generated in real time and no member of the research team saw or influenced the sequence, allocation concealment was complete by design; there was no possibility of foreknowledge or selective assignment.

12a) CONSORT: Statistical methods used to compare groups for primary and secondary outcomes

"All stimuli and data are available on the Open Science Framework (OSF)"

12a-i) Imputation techniques to deal with attrition / missing values

12b) CONSORT: Methods for additional analyses, such as subgroup analyses and adjusted analyses

The eight stimuli were constructed to be closely parallel. All presented the same core cancer prevention and genetic risk chatbot scenario in the same format and at comparable length, and differed only in (a) the normative frame (self-, peer-, expert-, or family-oriented) and (b) the one-sentence description of chatbot type. All stimulus texts are posted on OSF for direct comparison.

RESULTS

13a) CONSORT: For each group, the numbers of participants who were randomly assigned, received intended treatment, and were analysed for the primary outcome

13b) CONSORT: For each group, losses and exclusions after randomisation, together with reasons

Additional analyses were: (1) Bonferroni-corrected post hoc comparisons among the four norm message groups for each outcome with a significant main effect (Supplementary Table 1); (2) simple-effects comparisons of chatbot type within each norm message condition to interpret the significant multivariate interaction; and (3) a sensitivity regression adding demographic controls: "To verify that these associations were not attributable to demographic differences, we re-estimated the model with demographic controls (age, gender, race, and education) entered in a prior block... Full results are reported in Supplementary Table 2." Confirmatory and exploratory analyses are explicitly distinguished in the Data analysis section.		
**13b-i) Attrition diagram**		
**14a) CONSORT: Dates defining the periods of recruitment and follow-up**		
N=1000 participants were randomized across the eight conditions and all 1000 received the assigned stimulus and were analyzed. Per-condition numbers (Figure 3): self-oriented x AI-powered n=129; self-oriented x rule-based n=120; expert-oriented x AI-powered n=119; expert-oriented x rule-based n=121; family-oriented x AI-powered n=126; family-oriented x rule-based n=131; peer-oriented x AI-powered n=121; peer-oriented x rule-based n=133. Marginal totals: self-oriented n=249, expert-oriented n=240, family-oriented n=257, peer-oriented n=254; AI-powered n=495, rule-based n=505.		
**14a-i) Indicate if critical "secular events" fell into the study period**		
**14b) CONSORT: Why the trial ended or was stopped (early)**		
There were no losses or exclusions after randomization. All 1000 randomized participants completed the post-exposure measures and were analyzed, as shown in the participant flow diagram (Figure 3: "Randomized (N = 1000)" to "Completed the survey and analyzed (N = 1000)"). As noted in Figure 3, "The number of panelists invited, screened, or excluded prior to delivery of the final matched sample was determined by the survey vendor (YouGov) and was not available to the investigators."		
**15) CONSORT: A table showing baseline demographic and clinical characteristics for each group**		
"Participants were recruited in October 2025 from a nationally representative sample using the YouGov panel." Recruitment, exposure, and outcome assessment occurred within that single month, in one session per participant; there was no follow-up period.		
**15-i) Report demographics associated with digital divide issues**		
**16a) CONSORT: For each group, number of participants (denominator) included in each analysis and whether the analysis was by original assigned groups**		
**16-i) Report multiple "denominators" and provide definitions**		
**16-ii) Primary analysis should be intent-to-treat**		
Only one denominator applies: all 1000 randomized participants completed the outcome measures and were included in every analysis. Because there was no system to use, no participation thresholds, no "users vs starters" distinction, and no dose-based subgroups exist. Regression models were estimated on N=1000, F(9, 990) = 113.41.		
**17a) CONSORT: For each primary and secondary outcome, results for each group, and the estimated effect size and its precision (such as 95% confidence interval)**		
The study ended when the pre-specified sample of N=1000 matched, weighted respondents was delivered by the survey vendor. It was not stopped early and no stopping rule was invoked.		
**17a-i) Presentation of process outcomes such as metrics of use and intensity of use**		
**17b) CONSORT: For binary outcomes, presentation of both absolute and relative effect sizes is recommended**		
"Details about participant characteristics can be found in Table 1." Summarized in the Participants section: "Participants (N = 1,000) ranged in age from 18 to 91 years (M = 48.44, SD = 17.76). The sample was approximately balanced by gender, with 48.6% (n = 486) identifying as male, and 37.4% (n = 374) reporting a high school education or less. The racial and ethnic composition was predominantly White (63.7%), followed by Hispanic (17.6%) and Black (11.6%)." No clinical characteristics were collected, as participants were a general population sample rather than a patient population.		
**18) CONSORT: Results of any other analyses performed, including subgroup analyses and adjusted analyses, distinguishing pre-specified from exploratory**		
Multivariate and univariate results are reported with effect sizes: "The main effect of the social norm message was significant, Pillai's trace = .103, F(30, 3156) = 3.73, P< .001, partial eta-squared = .034. The main effect of chatbot type was also significant, Pillai's trace = .028, F(10, 1050) = 3.02, P< .001, partial eta-squared = .028. Furthermore, a significant multivariate interaction effect was observed, Pillai's trace = .109, F(30, 3156) = 3.98, P< .001, partial eta-squared = .036." Group means and SDs by condition are in Table 2; estimated marginal means with 95% confidence intervals are plotted in Figures 3 and 4; Bonferroni-adjusted mean differences with exact P values are in Supplementary Table 1. For the primary outcome, the regression model reports standardized coefficients and P values: perceived benefits (beta = .253, P< .001), trust (beta = .33, P< .001), privacy concern (beta = -.11, P< .001), with R-squared = .508.		
**18-i) Subgroup analysis of comparing only users**		
**19) CONSORT: All important harms or unintended effects in each group**		
Not applicable. All outcomes were continuous multi-item scale scores analyzed with MANOVA and linear regression; no binary or dichotomous outcomes were defined, so risk ratios and risk differences do not apply. Effect sizes are reported as partial eta-squared, mean differences, and standardized regression coefficients.		
**19-i) Include privacy breaches, technical problems**		
**19-ii) Include qualitative feedback from participants or observations from staff/researchers**		
DISCUSSION		
**20) CONSORT: Trial limitations, addressing sources of potential bias, imprecision, multiplicity of analyses**		
**20-i) Typical limitations in ehealth trials**		
**21) CONSORT: Generalisability (external validity, applicability) of the trial findings**		
**21-i) Generalizability to other populations**		
**21-ii) Discuss if there were elements in the RCT that would be different in a routine application setting**		
**22) CONSORT: Interpretation consistent with results, balancing benefits and harms, and considering other relevant evidence**		
**22-i) Restate study questions and summarize the answers suggested by the data, starting with primary outcomes and process outcomes (use)**		
**22-ii) Highlight unanswered new questions, suggest future research**		
The Discussion opens by restating the aim and answering it: "This study advances research on digital health adoption by proposing and empirically testing HTAM, an integrative framework that bridges technology acceptance and health behavior perspectives... the findings demonstrate that both normative message framing and chatbot type significantly shape users' motivational and acceptance-related beliefs." For the primary outcome: "Peer-oriented messages consistently strengthened descriptive norms, injunctive norms, self-efficacy, trust, and usage intention," and "Participants in the AI-powered condition reported higher trust and usage intention." Null findings are addressed directly: "The non-significant effects of ease of use and self-efficacy in the regression model suggest that basic usability may function as a threshold condition rather than a primary driver of intention." Process/use outcomes are not applicable in this design and this is stated as a limitation.		
Other information		
**23) CONSORT: Registration number and name of trial registry**		
Post hoc and interaction analyses are reported in the Results and Supplementary Table 1, and the demographic-adjusted regression in Supplementary Table 2: "we re-estimated the model with demographic controls (age, gender, race, and education) entered in a prior block. Demographics alone explained little variance in usage intention (R-squared = .017), and the HTAM coefficients remained substantively unchanged when demographics were included." The confirmatory/exploratory boundary is pre-specified in Data analysis: hypothesis tests for H1 and H2 and the focal effects on usage intention are confirmatory; univariate effects on the remaining nine dependent variables are exploratory. No unplanned subgroup analyses by demographic group were conducted, and the Limitations note that "this study did not systematically examine demographic moderation effects."		
**24) CONSORT: Where the full trial protocol can be accessed, if available**		

No harms or unintended effects were observed or reported. The study involved a single exposure to brief, non-personalized written health information and a set of attitudinal questions, and was approved as minimal-risk research by the University of Utah IRB (IRB_00186651). No adverse events, distress reports, or participant complaints were recorded. One finding worth flagging for design practice, though not a harm to participants, is that heightened perceived severity was associated with lower usage intention (beta = -.047, P= .049), suggesting that fear-based framing in such chatbots "may inadvertently depress use."		
**25) CONSORT: Sources of funding and other support (such as supply of drugs), role of funders**		
This study was not registered in a clinical trial registry. It was a single-session message experiment testing a theoretical model of technology acceptance, involving no clinical intervention, no health outcome, and no patient population, and therefore fell outside the scope of ICMJE clinical trial registration. The study was reviewed and approved by the University of Utah IRB (IRB_00186651), and all stimuli and data are publicly available on the Open Science Framework.		
**X26-i) Comment on ethics committee approval**		
**x26-ii) Outline informed consent procedures**		
**X26-iii) Safety and security procedures**		
**X27-i) State the relation of the study team towards the system being evaluated**		
