# Supplementary material for: The Impact of Chatbot Type and Normative Messaging on Chatbot Usage Intention Based on the Health Technology Acceptance Model: Randomized Controlled Trial
Source: J Med Internet Res. 2026 Sep 15;28:e97773. doi: 10.2196/97773 (PMC13577661; doi:10.2196/97773)
Supplement: Supplementary file 2 [file jmir-v28-e97773-s001.docx]

**Supplementary Table 1.**

**Pairwise Comparisons of Social Norm Messages**

| **Dependent Variable** | **Comparison** | **Mean Difference (SE)** | **95% CI** |
| --- | --- | --- | --- |
| Descriptive Norm | Self – Peer | -0.20* (0.07) | -0.3710, -0.0230 |
|  | Self – Expert | 0.04 (0.07) | -0.1320, 0.2130 |
|  | Self – Family | -0.01 (0.06) | -0.1700, 0.1600 |
|  | Peer – Self | 0.20* (0.07) | 0.0230, 0.3710 |
|  | Peer – Expert | 0.24** (0.07) | 0.0620, 0.4130 |
|  | Peer – Family | 0.19* (0.06) | 0.0240, 0.3600 |
|  | Expert – Self | -0.04 (0.07) | -0.2130, 0.1320 |
|  | Expert – Peer | -0.24** (0.07) | -0.4130, -0.0620 |
|  | Expert – Family | -0.05 (0.06) | -0.2120, 0.1210 |
|  | Family – Self | 0.01 (0.06) | -0.1600, 0.1700 |
|  | Family – Peer | -0.19* (0.06) | -0.3600, -0.0240 |
|  | Family – Expert | 0.05 (0.06) | -0.1210, 0.2120 |
| Injunctive Norm | Self – Peer | -0.16* (0.05) | -0.2970, -0.0290 |
|  | Self – Expert | 0.00 (0.05) | -0.1300, 0.1360 |
|  | Self – Family | -0.01 (0.05) | -0.1360, 0.1180 |
|  | Peer – Self | 0.16* (0.05) | 0.0290, 0.2970 |
|  | Peer – Expert | 0.17** (0.05) | 0.0320, 0.3010 |
|  | Peer – Family | 0.15* (0.05) | 0.0250, 0.2830 |
|  | Expert – Self | -0.00 (0.05) | -0.1360, 0.1300 |
|  | Expert – Peer | -0.17** (0.05) | -0.3010, -0.0320 |
|  | Expert – Family | -0.01 (0.05) | -0.1410, 0.1160 |
|  | Family – Self | 0.01 (0.05) | -0.1180, 0.1360 |
|  | Family – Peer | -0.15* (0.05) | -0.2830, -0.0250 |
|  | Family – Expert | 0.01 (0.05) | -0.1160, 0.1410 |
| Perceived Benefit | Self – Peer | 0.03 (0.07) | -0.1550, 0.2200 |
|  | Self – Expert | 0.10 (0.07) | -0.0860, 0.2870 |
|  | Self – Family | 0.06 (0.07) | -0.1210, 0.2350 |
|  | Peer – Self | -0.03 (0.07) | -0.2200, 0.1550 |
|  | Peer – Expert | 0.07 (0.07) | -0.1210, 0.2570 |
|  | Peer – Family | 0.03 (0.07) | -0.1560, 0.2050 |
|  | Expert – Self | -0.10 (0.07) | -0.2870, 0.0860 |
|  | Expert – Peer | -0.07 (0.07) | -0.2570, 0.1210 |
|  | Expert – Family | -0.04 (0.07) | -0.2230, 0.1360 |
|  | Family – Self | -0.06 (0.07) | -0.2350, 0.1210 |
|  | Family – Peer | -0.03 (0.07) | -0.2050, 0.1560 |
|  | Family – Expert | 0.04 (0.07) | -0.1360, 0.2230 |
| Perceived Susceptibility | Self – Peer | -0.17 (0.09) | -0.4060, 0.0660 |
|  | Self – Expert | -0.04 (0.09) | -0.2720, 0.1970 |
|  | Self – Family | 0.01 (0.09) | -0.2140, 0.2340 |
|  | Peer – Self | 0.17 (0.09) | -0.0660, 0.4060 |
|  | Peer – Expert | 0.13 (0.09) | -0.1060, 0.3700 |
|  | Peer – Family | 0.18 (0.09) | -0.0480, 0.4070 |
|  | Expert – Self | 0.04 (0.09) | -0.1970, 0.2720 |
|  | Expert – Peer | -0.13 (0.09) | -0.3700, 0.1060 |
|  | Expert – Family | 0.05 (0.09) | -0.1780, 0.2740 |
|  | Family – Self | -0.01 (0.09) | -0.2340, 0.2140 |
|  | Family – Peer | -0.18 (0.09) | -0.4070, 0.0480 |
|  | Family – Expert | -0.05 (0.09) | -0.2740, 0.1780 |
| Perceived Severity | Self – Peer | -0.07 (0.05) | -0.2160, 0.0690 |
|  | Self – Expert | 0.10 (0.05) | -0.0410, 0.2420 |
|  | Self – Family | -0.01 (0.05) | -0.1450, 0.1260 |
|  | Peer – Self | 0.07 (0.05) | -0.0690, 0.2160 |
|  | Peer – Expert | 0.17* (0.05) | 0.0300, 0.3170 |
|  | Peer – Family | 0.06 (0.05) | -0.0730, 0.2010 |
|  | Expert – Self | -0.10 (0.05) | -0.2420, 0.0410 |
|  | Expert – Peer | -0.17* (0.05) | -0.3170, -0.0300 |
|  | Expert – Family | -0.11 (0.05) | -0.2460, 0.0270 |
|  | Family – Self | 0.01 (0.05) | -0.1260, 0.1450 |
|  | Family – Peer | -0.06 (0.05) | -0.2010, 0.0730 |
|  | Family – Expert | 0.11 (0.05) | -0.0270, 0.2460 |
| Ease of Use | Self – Peer | 0.04 (0.06) | -0.1310, 0.2040 |
|  | Self – Expert | 0.10 (0.06) | -0.0670, 0.2660 |
|  | Self – Family | 0.05 (0.06) | -0.1050, 0.2130 |
|  | Peer – Self | -0.04 (0.06) | -0.2040, 0.1310 |
|  | Peer – Expert | 0.06 (0.06) | -0.1060, 0.2320 |
|  | Peer – Family | 0.02 (0.06) | -0.1440, 0.1790 |
|  | Expert – Self | -0.10 (0.06) | -0.2660, 0.0670 |
|  | Expert – Peer | -0.06 (0.06) | -0.2320, 0.1060 |
|  | Expert – Family | -0.05 (0.06) | -0.2060, 0.1150 |
|  | Family – Self | -0.05 (0.06) | -0.2130, 0.1050 |
|  | Family – Peer | -0.02 (0.06) | -0.1790, 0.1440 |
|  | Family – Expert | 0.05 (0.06) | -0.1150, 0.2060 |
| Self-Efficacy | Self – Peer | -0.24*** (0.06) | -0.4090, -0.0750 |
|  | Self – Expert | -0.02 (0.06) | -0.1830, 0.1500 |
|  | Self – Family | -0.01 (0.06) | -0.1670, 0.1500 |
|  | Peer – Self | 0.24*** (0.06) | 0.0750, 0.4090 |
|  | Peer – Expert | 0.23* (0.06) | 0.0570, 0.3940 |
|  | Peer – Family | 0.23*** (0.06) | 0.0720, 0.3950 |
|  | Expert – Self | 0.02 (0.06) | -0.1500, 0.1830 |
|  | Expert – Peer | -0.23* (0.06) | -0.3940, -0.0570 |
|  | Expert – Family | 0.01 (0.06) | -0.1520, 0.1690 |
|  | Family – Self | 0.01 (0.06) | -0.1500, 0.1670 |
|  | Family – Peer | -0.23*** (0.06) | -0.3950, -0.0720 |
|  | Family – Expert | -0.01 (0.06) | -0.1690, 0.1520 |
| Privacy Concern | Self – Peer | 0.58*** (0.09) | 0.3440, 0.8120 |
|  | Self – Expert | 0.19 (0.09) | -0.0400, 0.4250 |
|  | Self – Family | 0.42*** (0.08) | 0.2000, 0.6450 |
|  | Peer – Self | -0.58*** (0.09) | -0.8120, -0.3440 |
|  | Peer – Expert | -0.39*** (0.09) | -0.6210, -0.1490 |
|  | Peer – Family | -0.16 (0.09) | -0.3810, 0.0710 |
|  | Expert – Self | -0.19 (0.09) | -0.4250, 0.0400 |
|  | Expert – Peer | 0.39*** (0.09) | 0.1490, 0.6210 |
|  | Expert – Family | 0.23* (0.09) | 0.0060, 0.4550 |
|  | Family – Self | -0.42*** (0.08) | -0.6450, -0.2000 |
|  | Family – Peer | 0.16 (0.09) | -0.0710, 0.3810 |
|  | Family – Expert | -0.23* (0.09) | -0.4550, -0.0060 |
| Trust | Self – Peer | -0.20* (0.07) | -0.3740, -0.0200 |
|  | Self – Expert | -0.01 (0.07) | -0.1840, 0.1690 |
|  | Self – Family | -0.12 (0.06) | -0.2930, 0.0440 |
|  | Peer – Self | 0.20* (0.07) | 0.0200, 0.3740 |
|  | Peer – Expert | 0.19* (0.07) | 0.0110, 0.3680 |
|  | Peer – Family | 0.07 (0.07) | -0.0980, 0.2440 |
|  | Expert – Self | 0.01 (0.07) | -0.1690, 0.1840 |
|  | Expert – Peer | -0.19* (0.07) | -0.3680, -0.0110 |
|  | Expert – Family | -0.12 (0.06) | -0.2870, 0.0530 |
|  | Family – Self | 0.12 (0.06) | -0.0440, 0.2930 |
|  | Family – Peer | -0.07 (0.07) | -0.2440, 0.0980 |
|  | Family – Expert | 0.12 (0.06) | -0.0530, 0.2870 |
| Usage Intention | Self – Peer | -0.23 (0.11) | -0.5130, 0.0490 |
|  | Self – Expert | 0.07 (0.11) | -0.2060, 0.3530 |
|  | Self – Family | -0.22 (0.10) | -0.4900, 0.0440 |
|  | Peer – Self | 0.23 (0.11) | -0.0490, 0.5130 |
|  | Peer – Expert | 0.31* (0.11) | 0.0220, 0.5890 |
|  | Peer – Family | 0.01 (0.10) | -0.2630, 0.2790 |
|  | Expert – Self | -0.07 (0.11) | -0.3530, 0.2060 |
|  | Expert – Peer | -0.31* (0.11) | -0.5890, -0.0220 |
|  | Expert – Family | -0.30* (0.10) | -0.5660, -0.0280 |
|  | Family – Self | 0.22 (0.10) | -0.0440, 0.4900 |
|  | Family – Peer | -0.01 (0.10) | -0.2790, 0.2630 |
|  | Family – Expert | 0.30* (0.10) | 0.0280, 0.5660 |

*Note:* SE = standard error; CI = confidence interval. Significance levels: **p* < .05, ***p* < .01, ****p* < .001 (Bonferroni-adjusted).
