# Supplementary material for: The Impact of Chatbot Type and Normative Messaging on Chatbot Usage Intention Based on the Health Technology Acceptance Model: Randomized Controlled Trial
Source: J Med Internet Res. 2026 Sep 15;28:e97773. doi: 10.2196/97773 (PMC13577661; doi:10.2196/97773)
Supplement: Supplementary file 3 [file jmir-v28-e97773-s002.docx]

**Supplementary Table 2.** *Hierarchical Regression Predicting Usage Intention with Demographic Controls*

| **Predictor** | **Model 1** | | **Model 2** | | **Model 3** | |
| --- | --- | --- | --- | --- | --- | --- |
|  | ***β*** | ***p*** | ***β*** | ***p*** | ***β*** | ***p*** |
| ***Demographic controls*** | | | | | | |
| Age | -.118 | <.001 | .030 | .230 | .031 | .192 |
| Gender | -.018 | .573 | .029 | .230 | .018 | .419 |
| Race | .029 | .364 | .074 | .003 | .074 | .001 |
| Education | .037 | .241 | -.019 | .447 | .036 | .126 |
| ***Health motivation*** | | | | | | |
| Descriptive norm |  |  | .158 | <.001 | .091 | .002 |
| Injunctive norm |  |  | .096 | .002 | .075 | .012 |
| Perceived susceptibility |  |  | .046 | .065 | .053 | .022 |
| Perceived severity |  |  | -.080 | .002 | -.051 | .033 |
| Perceived benefit |  |  | .517 | <.001 | .251 | <.001 |
| ***Technology acceptance*** | | | | | | |
| Ease of use |  |  |  |  | .059 | .075 |
| Self-efficacy |  |  |  |  | -.047 | .098 |
| Trust |  |  |  |  | .341 | <.001 |
| Privacy concern |  |  |  |  | -.104 | <.001 |
| **R²** | .017 | | .422 | | .515 | |
| **ΔR²** | .017 | | .405 | | .093 | |
| **Adjusted R²** | .013 | | .417 | | .509 | |

*Note. β* = Standardized coefficients. *N = 1000. * p < .05, ** p < .01, *** p < .001.*
